# Rendezvous intervention using combined surgical carotid endarterectomy followed by endovascular thrombectomy in patients with acute tandem occlusions: a proof-of-concept experience at a tertiary care center

**DOI:** 10.1186/s42466-023-00290-4

**Published:** 2023-12-07

**Authors:** Norma J. Diel, Stefan T. Gerner, Omar Alhaj Omar, Johannes Kalder, Enikö Manz, Paula R. Keschenau, Tobias Struffert, Thomas Brueckner, Hagen B. Huttner, Thorsten R. Doeppner

**Affiliations:** 1 Department of Neurology, University Hospital Giessen, Klinikstrasse 33, 35392 Giessen, Germany; 2 Center for Mind, Brain and Behavior (CMBB), University of Marburg, Marburg, Germany; 3 Department of Adult and Pediatric Cardiovascular Surgery, University Hospital Giessen, Giessen, Germany; 4 Department of Neuroradiology, University Hospital Giessen, Giessen, Germany

**Keywords:** Tandem occlusion, Rescue therapy, Endovascular thrombectomy, Carotid endarterectomy

## Abstract

**Background:**

Endovascular thrombectomy (EVT) is highly effective in acute stroke patients with intracranial large vessel occlusion (LVO), however, presence of concomitant cervical occlusion of the internal carotid artery (ICA) may limit the endovascular access. This study describes feasibility and efficacy of a surgical carotid access (cutdown) to perform interdisciplinary recanalization therapy including carotid endarterectomy (CEA) followed by EVT for recanalization of intracranial LVO in stroke patients with tandem occlusions.

**Methods:**

We identified stroke patients with tandem occlusions who underwent a combined surgical-endovascular approach over a 5-year period. Surgical cutdown was provided by a cardiovascular surgery team at the angio-suite followed by EVT performed by the neuroradiological team. Demographics, stroke characteristics, treatments including antithrombotic management, procedure times, and clinical follow-up were assessed.

**Results:**

Four patients with acute stroke because of tandem occlusions received CEA followed by EVT (two patients after frustrating femoral catheterization, two as first-line approach). Successful recanalization (TICI ≥ 2b) via endovascular thrombectomy was achieved in all patients at a median of 28 min after successful surgical CEA. Intraprocedural complication was observed in one case (25%; i.e. ICA dissection).

**Conclusions:**

This small study provides evidence that a combined interdisciplinary approach of CEA followed by EVT in the angio-suite in acute stroke patients with tandem occlusions is a feasible procedure in patients otherwise not accessible to endovascular recanalizing therapy and, therefore, high likelihood of developing large hemispheric infarction. Prospective data are warranted to identify patients who benefit from this combined approach as first-line therapy.

## Background

Tandem occlusions of the internal carotid artery (ICA) and middle cerebral artery (MCA) represents a devastating subtype of acute ischemic stroke (AIS) associated with high morbidity and mortality [14]. Acute treatment of these patients reflects a complex and tricky endeavor and is mainly based on endovascular attempts to recanalize ICA followed by MCA [5]. While endovascular thrombectomy (EVT), with or without the addition of intravenous thrombolysis (IVT), is highly effective in AIS patients with intracranial large vessel occlusion (LVO) [6], its power in the aforementioned extreme scenarios involving both intracranial and extracranial vessels is less extensively studied [4]. Given that severe stenosis or occlusion of the cervical ICA limits endovascular access to intracranial LVO, recanalization of long-distance ICA occlusions, including rescue stenting, is required as a first line treatment [2]. However, this attempt, if accomplished at all, is often time-consuming and can lead to delays in intracranial EVT, both of which associated with poor patients’ clinical course [19]. Moreover, peri- and post-procedural clinical management is potentially harmful in light of a necessary intensified antithrombotic management, often including dual antiplatelet therapy and tirofiban [7].

To address this clinical dilemma, an alternative approach of a hybrid rendezvous intervention using surgical carotid endarterectomy (CEA) followed by EVT in these settings has been proposed recently [15, 16]. Yet, these reports rather focused on surgical and technical aspects than reporting clinical neurological data and stressing the potential implications for acute stroke management. Thus, uncertainty remains whether or not this approach is clinically valuable and feasible in daily routine [15, 16]. We here report a case series of acute stroke patients with ICA and MCA tandem occlusions who underwent—on an individual basis and as rescue intervention—combined revascularization of ICA and MCA by CEA followed by EVT within the angiography suite.

## Methods

All patients with combined CEA and EVT interventions within the angio-suite were identified from our Giessen stroke registry (GIST; ClinicalTrials.gov Identifier: NCT05295862) which includes all stroke patients treated at the University Hospital Giessen, Germany, over a 5 year-period. Demographics (age, sex), clinical and stroke characteristics (NIHSS, mode and time-point of recanalizing therapies), initial imaging parameters (site of LVO, ASPECT score) and functional outcomes (i.e. modified Rankin scale [mRS]) at discharge and 90 days were extracted from this institutional database. Further, we recorded the time-points of each procedural step in these patients, reviewed medical records and follow-up imaging for antiplatelet treatment regimes, occurrence of complications and final infarct size estimation using ASPECT scores.

Descriptive analyses were performed using SPSS 28.0 (www.ibm.com), presenting absolute numbers (percentages) and median (range) for relevant data in the overall cohort. Individual parameters for each case are reported separately.

## Results

Four acute stroke patients with tandem occlusions were treated with the combined interdisciplinary approach including surgical CEA and EVT between 03/2019 and 01/2023 (age range 69-86y; one female patient; see Table 1). Prior cardiovascular comorbidities were frequently present including arterial hypertension, diabetes, chronic kidney disease, coronary heart disease and previous strokes (Table 1). Previous stroke failure known severe ICA-stenosis in 3 of 4 patients (75%). All patients were severely affected (NIHSS 15–21) by AIS due to concomitant occlusion of cervical ICA and ipsilateral MCA at M1-level. Two out of four patients had left-hemispheric stroke, median ASPECT score on initial imaging was 7 (range 6–10).

**Table 1:** Patient and stroke characteristics

	Overall	Patient 1	Patient 2	Patient 3	Patient 4
Age (y)^+^	81 (69–86)	79	69	83	86
Female sex*	1 (25%)	–	–	Yes	–
Premorbid mRS (0–5) ^+^	1.5 (0–3)	1	3	2	0
*Stroke characteristics*					
Location of large vessel occlusion	n.a	R. ICA/M1	R. ICA (functional occlusion)/M1	L. ICA (functional occlusion)/M1/M2	L. ICA/M1
NIHSS (0–42)^+^	17.5 (15–21)	15	19	21	16
ASPECTS (0–10)^+^	7 (6–10)	6	6	10	8
Prior medication*					
Antiplatelet agents	3 (75%)	ASA + Clopidogrel	ASA	ASA	–
Statin	0 (0%)	–	–	–	–
Comorbidities*					
Arterial hypertension	4 (100%)	Yes	Yes	Yes	Yes
Coronary heart disease	3 (75%)	Yes	Yes	Yes	–
Atrial fibrillation	1 (25%)	–	–	Yes	–
Previous Stroke	1 (25%)	–	Yes	–	–
Chronic kidney disease/hemodialysis	3 (75%)/1 (25%)	Yes/–	Yes/–	Yes/yes	–
Peripheral arterial occlusive disease	1 (25%)	Yes	–	–	–
Known carotid artery stenosis	3 (75%)	Yes	Yes	Yes	–
Diabetes mellitus	1 (25%)	–	Yes	–	–
Hypercholesterolemia	2 (50%)	Yes	–	Yes	–

*n (%); ^+^median (range)

All four patients underwent combined interventional treatment under general anesthesia (Table 2). The indication for the combined treatment was made on an individual basis and consented among neurologists, neuroradiologists and cardiovascular surgeons.

**Table 2:** Revascularization therapy and procedural times

	Overall	Patient 1	Patient 2	Patient 3	Patient 4
Intravenous thrombolysis*	2 (50%)	Yes	–	–	Yes
Surgical procedure*	n.a	CEA + 6F carotid sheath	CEA	CEA + 6F carotid sheath	CEA + 6F carotid sheath
Direct surgical access*	2 (50%)	–	–	Yes	Yes
Aspiration catheter*	n.a	SOFIA	SOFIA	SOFIA	SOFIA
*Treatment time intervals* (hh:mm)^+^					
Onset-to-door	n.a	1:30	Wake-up	Inhospital	Inhospital
Door-to-image	0:17 (0:12–0:21)	0:21	0:12	n.a	n.a
Onset-to-image	0:24 (0:20–0:27)	n.a	n.a	0:20	0:27
Image-to-puncture/cutdown	1:09 (0:58–1:15)	1:03	1:15	1:15	0:58
Cutdown-to-recanalization	2:12 (1:22–3:01)	1:22	2:00	3:01	2:23
Detailed procedural times*, °, median (range)					
T0–T1	0:51 (0:39–1:02)	0:39	1:02	n.a	n.a
T1–T2	0:27 (0:23–0:31)	0:23	0:31	n.a	n.a
T2–T3	1:39 (1:01–2:34)	1:01	1:31	2:34	1:47
T3–T4	0:28 (0:21–0:36)	0:21	0:29	0:27	0:36
T4–T5	1:18 (0:48–1:41)	1:22	1:14	0:48	1:41
T0–T5	3:57 (3:46–4:47)	3:46	4:47	3:49	4:04

*n (%); ^+^median (range); °alternating leadership of neuroradiology (NR) and vascular surgery (VS) teams. T0–T1: initial catheterization through femoral artery attempted by NR. T1–T2: time delay between team takeovers. T2–T3: VS performing surgical access to carotid artery, endarterectomy of ICA and sheath implantation if needed. T3–T4: time from when NR took over again until successful endovascular thrombectomy and recanalization. T4–T5: time from recanalization to wound closure. T0–T5: total time between first cut or puncture (whatever came first) and wound closure (i.e. total duration of the rendezvous intervention)

### Unsuccessful endovascular approach and rescue crossover to rendezvous approach

**Fig. 1:**
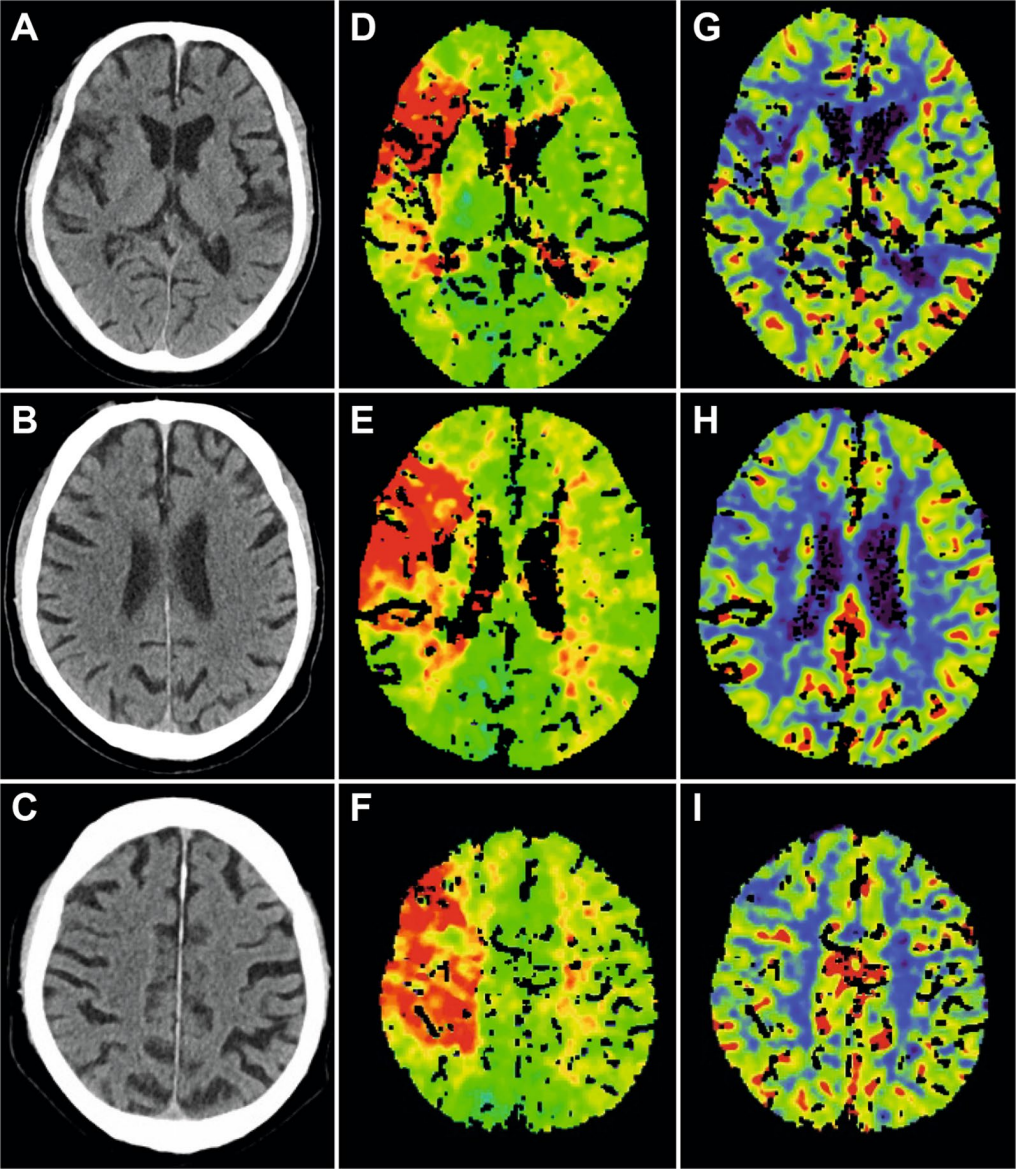
Initial imaging of patient 1. Non-contrast CT images (**A**–**C**) and CT-perfusion imaging including time to peak (TTP; **D**–**F**) and cerebral blood volume maps (CBV; **G**–**I**) on admission are provided. On initial imaging, minimal early ischemic changes were present (**A**–**C**), contrary to the large mismatch (TTP/CBV) detected by perfusion imaging (**D**–**I**)

**Fig. 2:**
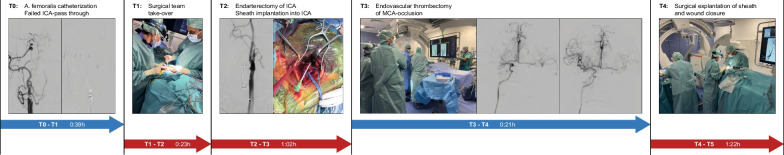
Step-by step illustration of the rendezvous approach. Illustration of the surgical and endovascular hybrid thrombectomy in patient 1. The blue timeline represents procedures performed by the neuroradiological team (NR), while the red one represents those performed by the vascular surgery team (VS). T0–T1: Initially, endovascular access was achieved by puncture of the femoral artery by NR. In the angiography, the internal carotid artery (ICA) did not show contrast enhancement (left) and several attempts to pass-through failed (right) prompting the involvement of the VS team after 0:39 h. T1–T2: The delay until takeover by VS lasted 0:23 h. T2–T3: Within 1:02 h, VS established the surgical access, performed a carotid endarterectomy of the ICA (left), and implanted a carotid sheath (right). T3–T4: NR took over again (left). Middle cerebral artery occlusion at the M1-level was still present (middle) and was recanalized by endovascular thrombectomy via the surgically implanted sheath (right; duration 0:21 h). T4–T5: After thrombectomy, the carotid sheath was removed and the wound was closed by VS (1:22 h). T0–T5: The entire procedure, from initial groin puncture to wound closure, lasted 3:46 h

Patients 1 and 2 presented with right-sided ICA occlusions; patient 1 received IVT within established time window, whereas patient 2 presented as wake-up stroke not eligible for IVT. Multimodal imaging confirmed indication for EVT (Fig. 1), groin puncture was performed 63 (patient 1) min, 75 (patient 2) min respectively, after imaging (Table 2). The endovascular approach proved unsuccessful to probe ICA why after 39 min, and 62 min respectively, a crossover to a combined treatment approach was decided (Fig. 2, Table 2). Vascular surgeons took over in the angio-suite and surgical cutdown was performed 23 min, 31 min respectively, later. CEA procedure including implantation of a 6F sheath into the ICA lasted for 61 min, 91 min respectively. Subsequently, the endovascular team performed EVT using a SOFIA aspiration catheter via the implanted sheath (Fig. 2, Table 2). After successful thrombectomy, cervical wound closure was performed by the vascular surgeon. Overall length of procedure was 3:46 h (T0–T5), and 4:47 h respectively (Fig. 2, Table 2).

### Direct rendezvous approach without endovascular attempt to recanalize ICA

Following up to both crossover patients reported above, two further patients with similar imaging patterns (patients 3 and 4, Tables 1, 2) received direct surgical cutdown 75 min (patient 3) and 58 min (patient 4) after imaging, respectively, skipping the initial endovascular approach of patients 1 and 2 attempting to recanalize ICA. In essence, indication for this direct combined approach was made as individual decisions in light of in-hospital strokes. Upon qualifying diagnostic imaging both interventional teams consented to perform the direct combined approach. Time saving of this direct rendezvous approach as compared to patients 1 and 2 was > 60 min.

### Clinical course and outcomes

**Fig. 3:**
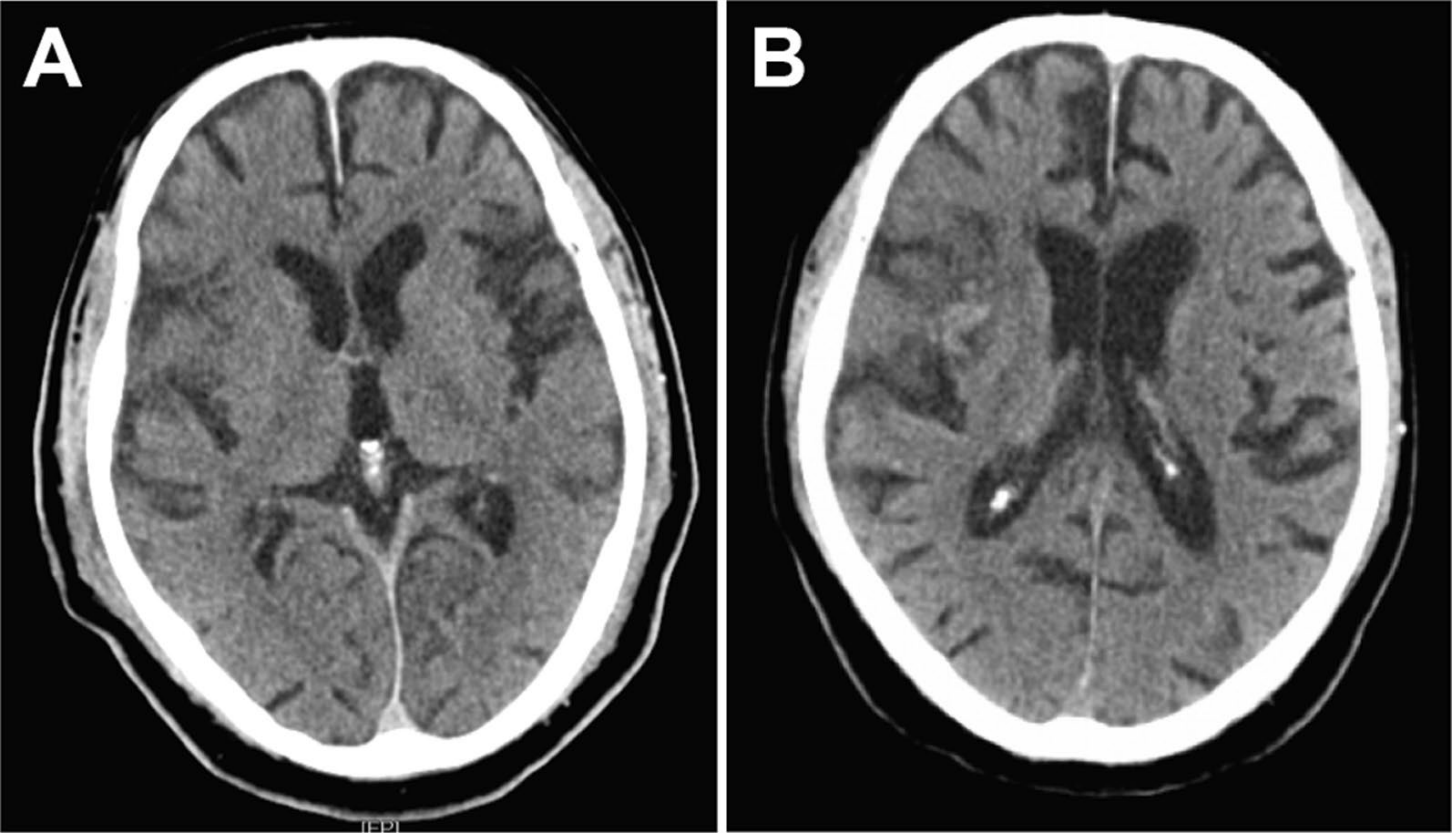
Radiological outcome in follow-up imaging. NCCT indicates non-contrast cranial CT. One day post intervention, only a small infarct demarcation was observed in the right insula (**A**) without any bleeding complication. Nine days post intervention, hemorrhagic transformation within the infarcted region became evident (**B**)

Efficacy and safety parameters are presented in Table 3. Successful recanalization (TICI ≥ 2b) was achieved in all patients. Only one relevant intraprocedural complication was reported, i.e. periprocedural dissection of the ICA requiring additional stenting (patient 3). Follow-up imaging 24 h after intervention revealed no significant increase of brain infarction compared to initial imaging (ASPECTS median 7.5; Fig. 3). Clinical course however was serious, with one patient developing multi organ failure leading to DNR/DNT orders and comfort care decisions. 90 day follow up of the other three patients revealed a mRS score of 4 in one patient, whereas the other two patients died after refusing life support including artificial respiration.

**Table 3:** Efficacy and safety outcomes

	Overall	Patient 1	Patient 2	Patient 3	Patient 4
TICI 2b/3*	4 (100%)	3	2b	2b	2b
Intraprocedural complications*	1 (25%)	–	–	–	ICA-dissection
Final infarct size (ASPECTS 0–10)^+^	7.5 (4–9)	9	8	4	7
Antiplatelet therapy*/starting day	3 (75%)	ASA + Clopidogrel/1d post-OP	ASA/4d post-OP	–	Tirofiban/periinterventional Clopidogrel/1d post-OP
Ventilation duration (h)^+^	265 (34–513)	513	235	34	295
Tracheotomy*	1 (25%)	Yes	–	–	–
Duration of ICU stay (d)^+^	13.5 (2–23)	23	13	2	14
Mortality at day 7*	1 (25%)	–	–	Yes	–
mRS at day 7^+^	5 (5–6)/6 (4–6)	5	5	6	5
Received DNT/DNR order	3 (75%)	No	Yes	No	Yes
mRS at 90 days (0–6)^+^	6 (4–6)	4	6	6	6

*n (%); ^+^median (range); DNT/DNR order: do-not-treat/do-not-resuscitate orders

## Discussion

This small study established feasibility and safety of a combined rendezvous approach within the angio-suite for surgical CEA to recanalize ICA, followed by EVT for revascularization of MCA in acute stroke patients. Several aspects emerge from the data.

Tandem occlusions affect approximately 10–15% of stroke patients and are specifically challenging [19]. Some advocate avoiding interventional treatments in such cases and relying solely on intravenous thrombolysis, yet successful reperfusion rate is likely to be insufficient in tandem occlusions [14]. A meta-analysis has shown benefits of thrombectomy for this patient subset, yet uncertainty remains regarding optimal technique to achieve access to intracranial LVO [6, 8]. Specifically, in patients with pre-existing high-grade ICA stenosis, initial endovascular approach appears demanding.

As a consequence, as an alternative technical approach to conventional femoral catheterization for endovascular access to the extra- und intracranial vessels, a direct ultrasound-guided carotid puncture has been proposed [10]. However, this technique harbors some shortcomings, risk of dissection, individual anatomical difficulties as well as post-procedural complications in access site closure [13], all of which undermine its common application in acute stroke patients. Another option involves direct surgical access to the ICA, as demonstrated by vascular surgeons 2019 before the corona lock-down. This study demonstrated the feasibility of surgical access without significant procedural complications, even when systemic thrombolysis was administered concurrently [16]. Yet, scope of this publication were technical aspects and feasibility descriptions addressed at the surgical community without having achieved its well-deserved recognition and creditability among acute neurovascular stroke physicians. Therefore, the present study of a combined surgical and endovascular revascularization may now offer clinical and procedural management information to the community of clinical neurologists, highlighting the feasibility and safety of the combined approach for patients otherwise not treatable by a conventional endovascular approach.

Even if individual risk factors of acute stroke patients are known, the success of femoral access-based recanalization of ICA and MCA remains uncertain in each case. Therefore, one must carefully consider the risk of a futile catheterization attempt delaying recanalization versus the risks associated with a more invasive cutdown approach. In our report, initial cutdown would have saved more than 60 min in those patients who crossed-over after unsuccessful endovascular approach. An additional potential benefit of the combined approach is the lack of ICA stenting (anterograde, stenting first or retrograde, thrombectomy first) performed otherwise during the endovascular approach [19]. As stenting requires dual antiplatelet treatment the subsequent risk of intracranial hemorrhage is increased, specifically in larger infarct volumes [7, 9]. The discussion to prefer balloon catheterization (angioplasty) alone followed by thrombectomy, leaving stenting for a second intervention at a later stage [1], is currently not recommended based on all evidence in light of a higher risk of stroke recurrence and progression rate [18]. Hence, the combined approach with initial surgical cutdown opens up a valuable and clinically feasible avenue, allowing direct CEA with subsequent EVT on a timely basis for patients with tandem occlusions.

Overall, patients with tandem occlusions have a poor prognosis why—in light of all aspects discussed above—interpretation of clinical outcomes in patients with tandem occlusions treated by one or the other approach is tricky [3, 18]. One important aspect refers to the fact that there have been no randomized clinical trials primarily focusing on tandem occlusion patients no far; existing evidence mainly was derived from post-hoc analyses of subsets of patients, why the first RCTs have been initiated such as the EASI-TOC (NCT04261478) and the TITAN (NCT03978988) trials. Hence, the outcomes described here, should not be overrated for the following reasons: patients have had most severe comorbidities prone to decompensation during protracted clinical course of treatment eventually resulting in care limitations and do-not treat orders. Moreover, the patients presented here represent a negative selection of patients presumably not having been eligible for recruitment into respective trials [11, 12]. Thus, even smaller series, specifically those which provide proof-of-principle data, appear valuable preceding subsequent trials [17].

Although the combined approach demonstrated recanalization in all patients in this study, it is important to consider limitations. The small number of cases and the lack of a control group preclude definitive conclusions regarding the superiority of this method over conventional endovascular approaches. Additionally, the decision to pursue a direct surgical cutdown should be carefully evaluated on an individual basis, as done here in those patients with in-hospital strokes only, weighing benefits and risks and taking into account individual risk factors, anatomical considerations, and available resources. Implementing such a collaborative framework requires significant organizational effort and a good understanding of each discipline's role in the procedure.

## Conclusions

In conclusion, this case series presents a combined treatment approach involving carotid endarterectomy and thrombectomy for acute stroke patients with tandem occlusions. The findings suggest that in cases where conventional endovascular access is not feasible, direct surgical cutdown may offer a viable alternative to achieve successful recanalization. However, due to the limited number of cases and the complex nature of these patients, cautious interpretation is necessary, and further research is required to determine the broader applicability and potential benefits of this approach in the management of tandem occlusions.

## Data Availability

All data generated or analysed during this study are included in this published article.

## Abbreviations

EVT: Endovascular thrombectomy
IVT: Intravenous thrombolysis
ICA: Internal carotid artery
CEA: Carotid endarterectomy
LVO: Large vessel occlusion
AIS: Acute ischemic stroke
NIHSS: National Institute of Health Stroke Scale
ASPECT: Alberta stroke program early CT score
TICI: Thrombolysis in cerebral infarction scale
mRS: Modified Rankin scale
